# Molecular engineering of polymersome surface topology

**DOI:** 10.1126/sciadv.1500948

**Published:** 2016-04-15

**Authors:** Lorena Ruiz-Pérez, Lea Messager, Jens Gaitzsch, Adrian Joseph, Ludovico Sutto, Francesco Luigi Gervasio, Giuseppe Battaglia

**Affiliations:** Department of Chemistry, University College London, 20 Gordon Street, WC1H 0AJ London, UK.

**Keywords:** Controlled self-assembly, block copolymers, vesicles, surface topology, biomimetic structures

## Abstract

Biological systems exploit self-assembly to create complex structures whose arrangements are finely controlled from the molecular to mesoscopic level. We report an example of using fully synthetic systems that mimic two levels of self-assembly. We show the formation of vesicles using amphiphilic copolymers whose chemical nature is chosen to control both membrane formation and membrane-confined interactions. We report polymersomes with patterns that emerge by engineering interfacial tension within the polymersome surface. This allows the formation of domains whose topology is tailored by chemical synthesis, paving the avenue to complex supramolecular designs functionally similar to those found in viruses and trafficking vesicles.

## Teaser

Self-assembling vesicles made of copolymer mimics biological systems.

## INTRODUCTION

Living systems are the result of a very precise and balanced hierarchical organization of molecules and macromolecules. These are constructed with specific chemical signatures that direct supramolecular interaction between themselves and/or with water. Such interactions, typically low in energy (that is, tens of *kT*s), allow the formation of mesoscale architectures with exquisite spatial and temporal control. This process, known as self-assembly, is ubiquitous in nature and is at the core of many biological transformations (*1*). Alongside positional self-assembly, nature creates energy gradients by enclosing chemicals into aqueous volumes using gated compartments (*2*). Both compartmentalization and positional self-assembly create structures whose surfaces express several chemistries performing their function holistically, according to specific topological interactions; for topology, here we refer to the arrangement of the various chemical components on a given surface. Biological surfaces are far from homogeneous systems and organize their components according to specific (quasi)regular patterns. It is well established that cell membranes have a mosaic-like structure made of dynamic nanoscale assemblies of lipids, sterols, glycols, and proteins collectively known as rafts, and that these rafts control membrane signaling and trafficking (*3*). Such a topological design is also found in smaller biological structures such as viruses, synaptic vesicles, lipoproteins, and bacteria. Structural subunits can be combined into topologies with supersymmetric arrangements, such as in most nonenveloped viruses (*4*), into semiordered topologies, such as in lipoproteins (*5*), or into Turing-like patterns as in most enveloped viruses (*6*) and endogenous trafficking vesicles (*7*). Surface topology is not stochastic and is the result of an evolutionary drive often associated with a specific function. Viruses, for example, change their surface topology during maturation from a noninfectious, almost inert assembly to an infectious cell-active structure capable of promptly entering cells (*8*). This would suggest that cellular targeting and signaling is not only controlled at a molecular level (that is, the single ligand-receptor interaction) but also at a mesoscale level (that is, how ligands and/or receptors are oraganized).

As our knowledge of this natural phenomenon advances, so do the efforts in creating functional materials and devices that use the same principles. Among the different biomimetic efforts, polymersomes are possibly one of the few examples that encompass both compartmentalization and positional self-assembly at the same time. Polymersomes are vesicles formed by the self-assembly of amphiphilic block copolymers in water. In analogy to natural phospholipids, polymersomes can house controlled aqueous volumes to create chemical potentials across the membranes (*9*). However, the macromolecular nature of the polymersome building blocks allows the design of vesicle membranes with control over their thickness, brush density, mechanical properties, and permeability (*10*). Furthermore, copolymers can be designed with tunable solubility, and hence, polymersomes can be made responsive to a large plethora of environmental stimuli such as pH, ionic strength, enzymatic degradation, hydrolysis, light, temperature, and many others (*11*–*13*). All these properties make polymersomes a very promising platform for drug and gene delivery with several examples of translation efforts of polymersomes into oncology, neurology, and immunology among others (*14*). More recently, we, along with others, have demonstrated that polymersomes can be designed with surface whose topology can be controlled by polymer/polymer interaction (*15*). The mixing of two partially immiscible polymersome-forming copolymers leads to the formation of vesicles whose surface can be patchy (binodal separation) or stripy (spinodal separation) (*16*). When the two copolymers have molecular mass mismatch, the same separation causes curvature instabilities, and thus the emergence of topographical features from the polymersome surface (*16*). We have demonstrated that topology has a great impact on how polymersomes interact with living cells, with the patchy configurations entering cells orders of magnitude more efficiently than the pristine ones (*17*). However, whether bimodal or spinodal, the separation leads to a full coarsening and the formation of fully asymmetric polymersomes over time (*15*). Here, we propose a new approach to control the polymersome topology using a membrane-confined self-assembly that creates the necessary interfacial energy to drive separation. We use molecules that can act as stabilizers decreasing the interfacial energy and hindering full phase separation. Such an entropic control over the final structure allows translation of positional self-assembly processes onto the polymersomes surface.

**Fig. 1:**
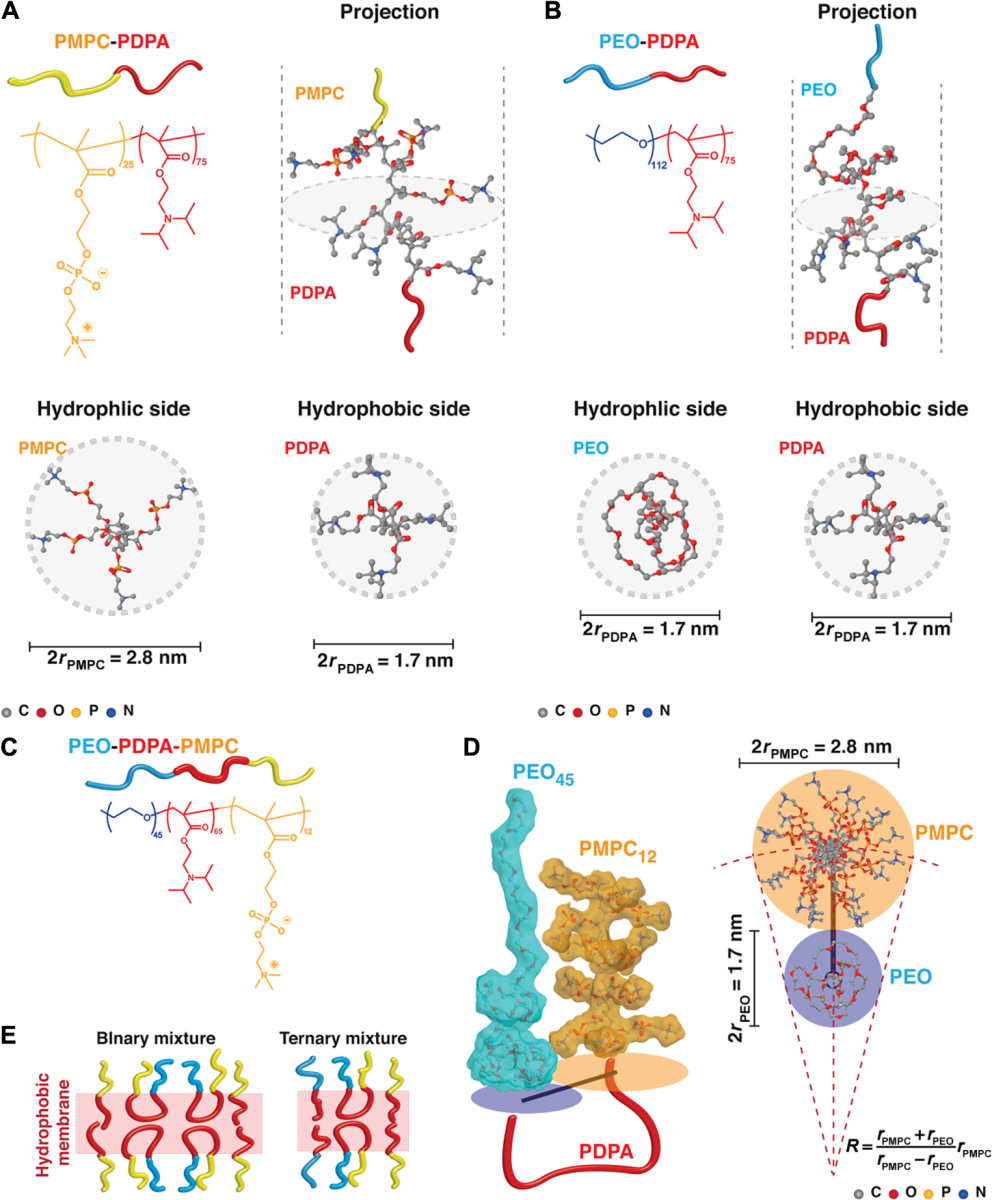
Copolymers’ chemical structure and conformation. (**A** and **B**) Molecular structure of PMPC-PDPA (A) and PEO-PDPA (B) with the corresponding molecular models showing the most probable configuration of the chains at the hydrophilic/hydrophobic interface. The models are shown as isometric projections and hydrophobic and hydrophilic views. The structure was minimized using the semiempirical method PM7. (**C** and **D**) Molecular structure of the PEO-PDPA-PMPC triblock (C) and the occupancy of the two hydrophilic block PEO and PMPC are calculated using the semiempirical method PM7 and represented as isometric projection and top view (D). (**E**) Possible arrangements of the triblock PEO-PDPA-PMPC in a binary mixture with PMPC-PDPA diblock and in a ternary mixture with PMPC-PDPA and PEO-PDPA diblocks.

We synthesized three different amphiphilic block copolymers based on poly(2-(diisopropylamino)ethyl methacrylate) (PDPA) hydrophobic block (Fig. 1). This system enables the formation of pH-sensitive polymersomes that can escape the endocytic degradation once internalized by cells (*18*). We combined hydrophobic PDPA with two biomedical relevant and biocompatible hydrophilic polymers, poly(2-methacryloyloxyethyl phosphorylcholine) (PMPC) and poly(ethylene oxide) (PEO), into two diblock copolymers, PMPC-PDPA and PEO-PDPA, and a linear triblock copolymer, PEO-PDPA-PMPC. Having demonstrated that PEO-PDPA and PMPC-PDPA copolymers can form patchy and/or stripy polymersomes (*17*), we introduce the triblock copolymer to control the phase separation between the PEO and PMPC blocks in all effect acting as a two-dimensional (2D) surfactant [also known as lineactant (*19*)]. In Fig. 1A, the structure of PMPC-PDPA is shown in addition to an optimized molecular model illustrating the spatial organization of the copolymer hydrophilic and hydrophobic segments at their interface, represented as isometric, hydrophilic-side, and hydrophobic-side views. PMPC_5_ and PEO_20_ were jointed together with PDPA_5_, and their structure was minimized using the semiempirical method PM7 (*20*) with the implicit solvent model COSMO (*21*) and a dielectric constant of 78.4 for the hydrophilic PMPC and 4.0 for the hydrophobic PDPA. Such an analysis allows assessment of how the two polymers behave at the hydrophobic/hydrophilic interface. For the PMPC-PDPA, it is evident that the bulky nature of the phosphorylcholine groups of the PMPC forces a larger area than that occupied by the PDPA units, and indeed more than sufficient to shield PDPA from water. Conversely, in PEO-PDPA copolymers, the ethylene oxide units are not sufficiently voluminous to cover the hydrophobic area of PDPA (Fig. 1C). This mismatch in area sizes imposes the PEO to collapse onto the PDPA area to prevent its contact with water. We reported a very similar behavior in other PEO-based polymersomes using small-angle x-ray scattering (SAXS) measurements (*22*). Our calculations estimated that at least 10 to 15 ethylene oxide (EO) units are required to cover the PDPA area with a mushroom-like configuration, in agreement with our previous SAXS measurements (*22*). However, we observed that the level of confinement of the PEO within the polymersome membrane still forces the rest of the chain into a stretched configuration (*22*). As for the PMPC, our model [as well as previous measurements we performed using advanced electron microscopy (*23*)] suggests that the PMPC chains will have interchain distances that are lower than the monomer size; hence, a fully stretched configuration is expected (*24*). Steric forces imposed by the phosphorylcholine groups also need to be taken into account in this system. Indeed, high steric forces have the capacity to hinder any chain coiling, supporting our suggestion of PEO and PMPC being fully stretched. In Fig. 1C, we show the structure of the PEO-PDPA-PMPC triblock copolymers, and using the above-mentioned considerations, we can estimate the triblock configuration when looped in the membrane, that is, with both PEO and PMPC facing the same side of the membrane (Fig. 1D). This allows us to estimate the occupancy of the two chains forced together to calculate a 2D packing factor. These structural considerations are critical to understanding how binary mixtures of PMPC-PDPA/PEO-PDPA-PMPC and ternary mixtures of PMPC-PDPA/PEO-PDPA-PMPC/PEO-PDPA copolymers assemble onto the polymersome surface.

## RESULTS AND DISCUSSION

**Fig. 2:**
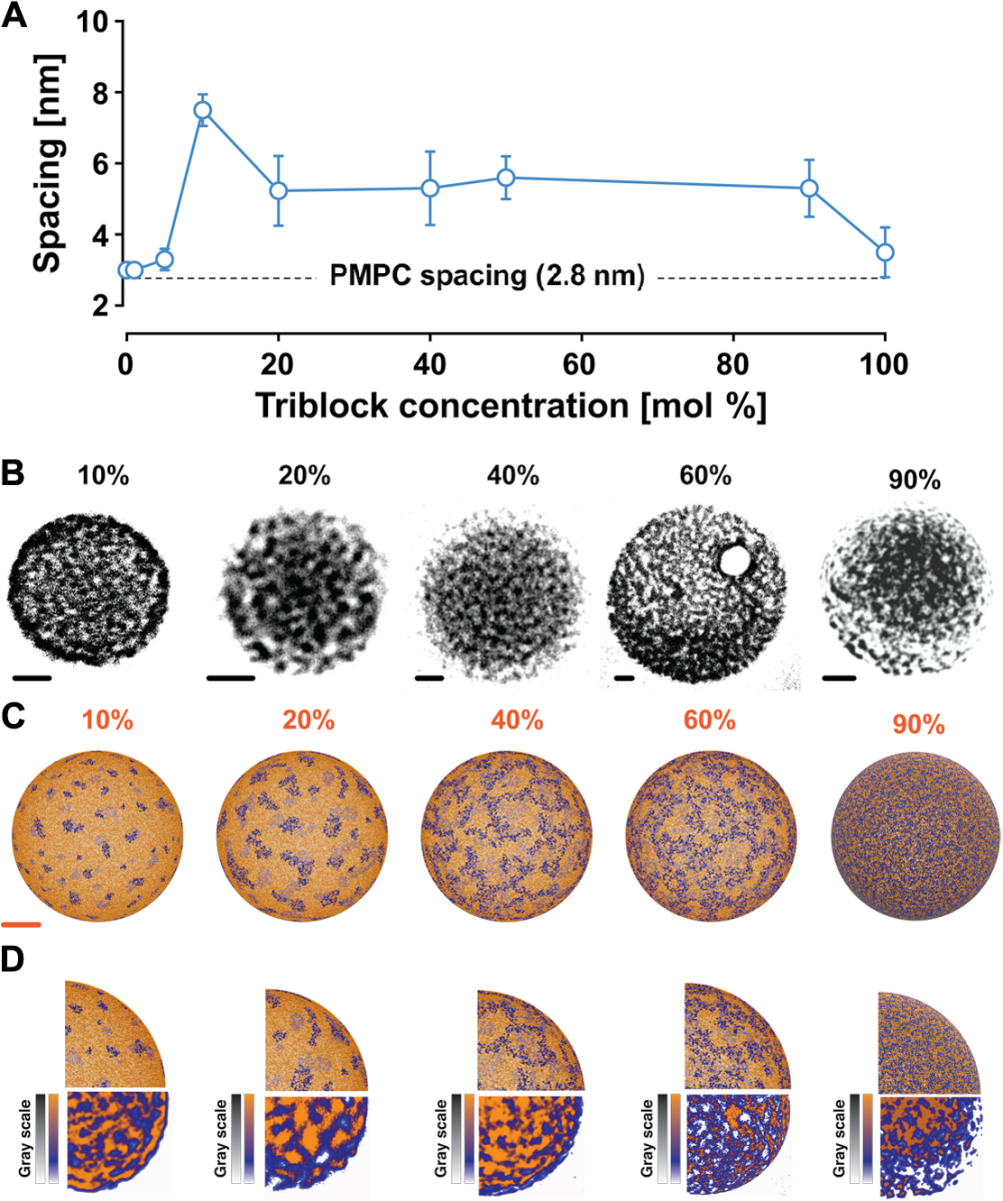
Binary phase diagram. (**A**) Graph showing the average spacing of the domains formed on the PMPC-PDPA/PEO-PDPA-PMPC polymersomes surface as a function of triblock concentration. (**B** and **C**) TEM images (B) and coarse-grained models (C) shown with semitransparent top surface to simulate transmission imaging of PMPC-PDPA/PEO-PDPA-PMPC polymersomes at different triblock concentrations. (**D**) Comparison of the polymersome surface patterns visualized by TEM and obtained by the simulations. The TEM images are shown using a color palette calibrated with the grayscale. Scale bars, 20 nm; gold is used to represent the PMPC domains, and blue to represent the PEO domains.

We previously demonstrated that phosphotungstic acid (PTA) can be used to highlight polymers that bear carboxylic groups, and by adjusting the contact time between the heavy metal and the dried polymersomes, we can distinguish between PMPC- and PEO-rich domains (*17*). Fortunately, the structure of the polymersome is quite robust, and it can survive controlled drying processes to allow dry state transmission electron microscopy (TEM). In these conditions, we can visualize the polymersome surface topology with high satisfactory spatial resolution and assess the effect of copolymer compositions and their ratios. In Fig. 2B, we show five micrographs illustrating the effect of triblock concentration in binary systems comprising PMPC-PDPA/PEO-PDPA-PMPC triblocks. In the first one, at 10% triblock concentration, several domains formed by the PEO chains are visible (white unstained PEO versus black stained PMPC). These domains have sizes ranging from 6 to 10 nm, with most of them having a circular shape and a few displaying a more elongated configuration. At higher concentrations, the elongated conformation becomes dominant, and at triblock concentrations between 40 and 80%, the domains merge, forming a bicontinuous pattern. Finally, at 90% concentration, the black domains seem to assume a discrete shape, suggesting some sort of symmetrical arrangement. Each formulation is analyzed by calculating an average spacing between the features visible on the polymersome surface and shown in Fig. 2A. The resulting graph and the average spacing do not change from ca. 3 nm from 0 to 10% of triblock concentrations. This is very similar to the dimension of a single PMPC chain, suggesting that either the triblock is dispersed homogeneously or the emergence of potential domains is not statistically significant at this concentration. At 10% triblock concentration, we have a considerable deviation with a spacing of ca. 7 nm. For higher concentrations, the spacing drops down to ca. 5 nm and stays constant for most triblock concentrations, with the exception of the 100% formulation where the spacing drops down to the single PMPC chain dimension. This finding suggests that the triblock copolymers form polymersomes with asymmetric membranes by itself, with either the outer or the inner layer exclusively expressing PMPC or PEO, respectively. This behavior is well established and was reported by Stoenescu *et al.* (*25*), and we also showed similar arrangements with PMPC-PDPA-PDMEA copolymers (*26*).

To rationalize the shape and pattern distribution observed experimentally on the polymersome surface, we devised a coarse-grained model of the copolymers diffusing on a spherical surface (see Materials and Methods). Each PMPC-PDPA copolymer is represented as a single bead of mass *m*_1_ representing the PMPC solvent-exposed chain. The total number of beads on the sphere surface was constant across the different simulations and resulted in *n* = 65539. To sample the equilibrium distribution of the beads on the sphere, we used the GROMACS molecular dynamics package (*27*) to perform 300-ns-long Langevin dynamics simulations of the system at increasing PEO-PDPA-PMPC/PMPC-PDPA ratios (5:95, 10:90, 20:80, 40:60, 50:50, 80:20, and 90:10), using an inverse friction constant *t* = 1 ps, an integration time step of 2 fs, and reference temperature *T* = 300 K. The resulting models are displayed as see-through transparent polymersomes, allowing a superposition view of the features present on both top and bottom surface areas of the sphere (Fig. 2C). This is performed as an effort to reproduce the polymersome projection images obtained from electron microscopy working in transmission mode. Indeed, we have no means to assess the opacity of the polymersome surfaces. Consequently, what we observe in the imaged structures might very well be superimposed stained features from the top and base of the polymersomes. In this fashion, rendering transparent the modeled polymersomes can compare them to experimentally imaged polymersomes. In Fig. 2C, we show that the corresponding results and the similarity between the simulation snapshot and the TEM micrographs are quite striking with a clear overlap of the two phases. Moreover, the shapes and pattern distribution obtained from the coarse-grained model on the polymersome surface mirror the same dependence on triblock concentration as that observed experimentally. In this fashion, at low concentrations of triblock, the blue (PEO) domains emerge to form isolated circular patches, which evolve into elongated shapes as triblock concentration increases in the copolymer mixture. The elongated conformations start to merge into bicontinuous patterns, and as triblock concentration increases, these patterns form a denser network. At 100% triblock concentration, the dense network covers the whole surface, forming the matrix with isolated orange (PMPC) domains as an inverted phase. Modeled and TEM-imaged polymersomes are directly compared in Fig. 2D.

**Fig. 3:**
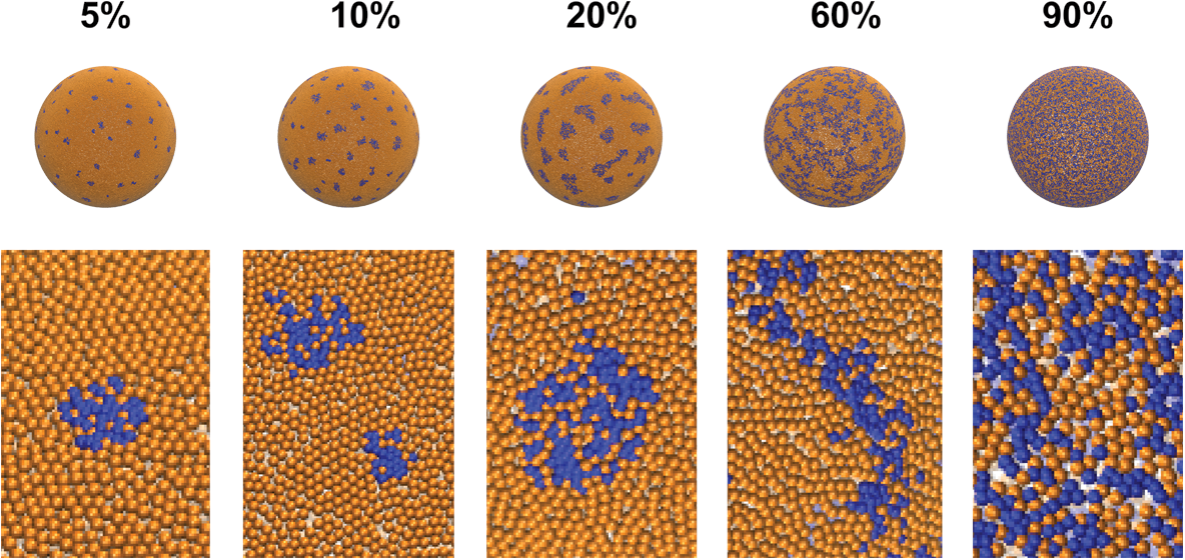
Coarse-grained simulation of PMPC-PDPA/PEO-PDPA-PMPC polymersomes at different triblock concentrations displayed with a nontransparent surface. Regions of interest extracted from the surface highlight our proposed mechanism of domain formation and its shape evolution as a function of triblock concentration. Gold is used to represent the PMPC domains, and blue to represent the PEO domains.

We can formalize these findings using the calculation for PMPC and PEO chain occupancy shown in Fig. 1F, suggesting a 2D micellization process of the triblock copolymer within a PMPC diblock matrix. The PMPC-PDPA-PEO triblock has a structural configuration that does not allow a perfect packing with an area mismatch between the two hydrophilic blocks of about 0.6. Hence, this area mismatch forbids the PEO chains to form regular hexagonal or triangular patterns perfectly surrounded by the PMPC chains. In Fig. 3, we show the arrangements using the top view of the modeled polymersomes, and these display the evolution of the triblock domain formation. At low concentration, the triblock copolymers form discrete noncircular domains (which we name 2D micelles), and these domains gradually evolve into more stripe-like structures as the triblock concentration increases, leading to the formation of bicontinuous surfaces. Such a process would explain the fact that the average spacing does not vary for a large range of triblock concentrations. Moreover, the 2D micellization process also suggests that we can organize the polymersome surface using the molecular design of the triblock as a building block.

**Fig. 4:**
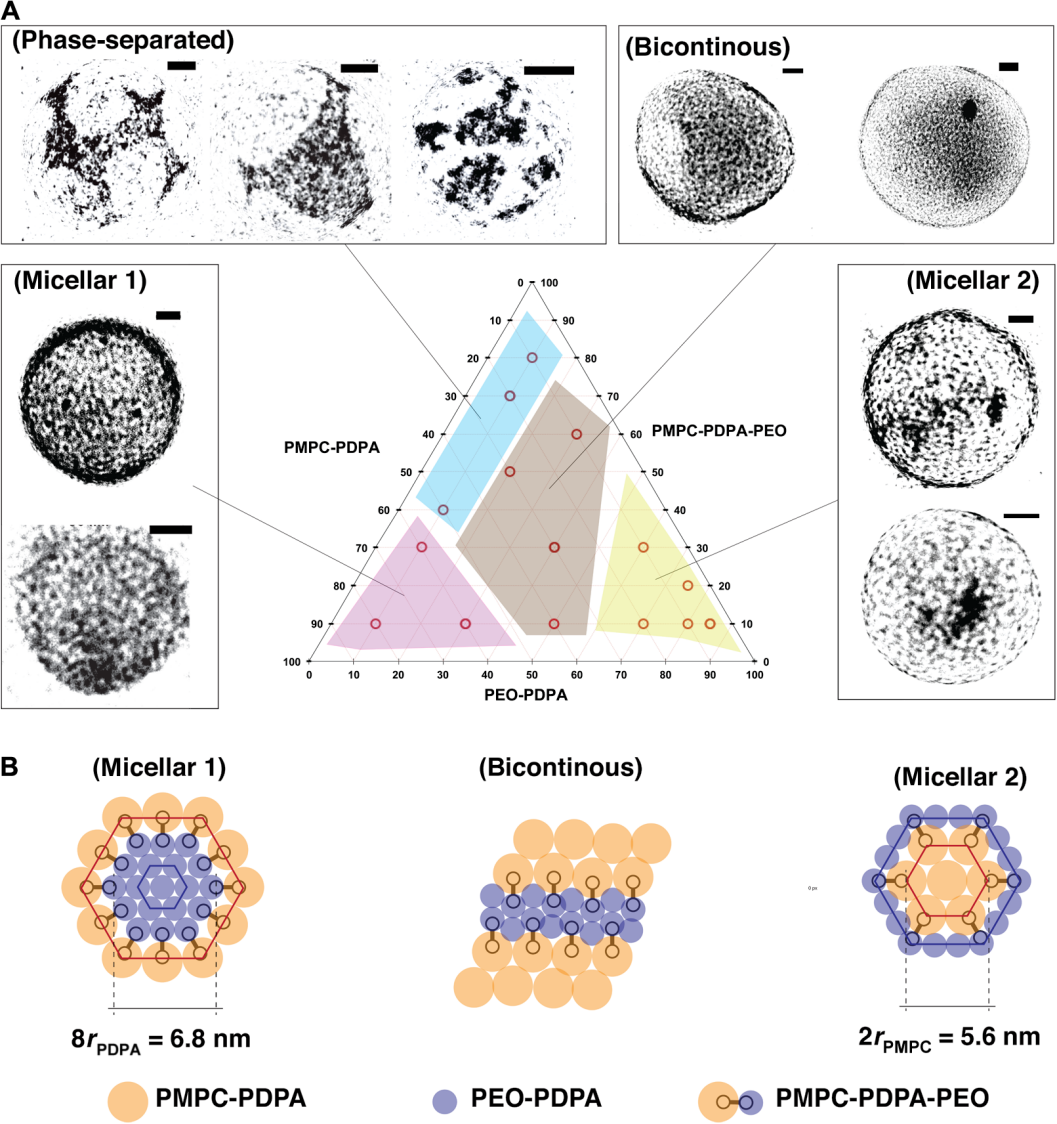
Ternary phase diagram. (**A**) Diagram of PMPC-PDPA/PEO-PDPA-PMPC/PEO-PDPA polymersomes. (**B**) Graphical representation of the three different phases observed in the diagram. Scale bars, 20 nm.

The molecular dimensions of the PMPC and PEO chains within the triblock are the critical parameters for controlling the triblock self-assembly on the polymersome membrane. Indeed, the results presented here propose some novel chemical design suggesting the creation of more asymmetrical configurations, that is, larger areas occupied by the PMPC chains when compared to PEO. The design of these asymmetrical configurations might very well lead to more ordered domains, such as those observed in viruses. However, a simpler approach to aid the self-assembly process can be achieved by adding PEO-PDPA copolymer in the system generating a ternary mixture. In Fig. 4A, we show the corresponding ternary diagram of the PMPC-PDPA/PEO-PDPA-PMPC/PEO-PDPA ternary polymersomes. The results are quite intriguing, and albeit more complex morphologies were expected, the diagram can be summarized into four different phases. At high concentrations of triblock and low concentrations of both diblocks (the top side of the diagram), we observed large domains on the polymersome surface with some level of symmetry but generally quite disordered, similar to the patterns displayed by uncontrolled diblock mixtures we previously reported (*16*). This might suggest the existence of a miscibility gap where the polymersome surface is formed by irregular domains with some internal orders within disordered PEO rich areas. The scenario is more symmetrical in the rest of the diagram where three very distinct phases can be identified. At high PMPC-PDPA concentrations (left-hand side of the diagram), we observed micellar phases with PEO domains (white) dispersed within a PMPC matrix (dark gray) with an average size of about 7 nm. These domains are highly convoluted, and although not quite apparent, most formulations show some sort of ordering of these 2D micelles arrangements. On the other extreme of the diagram (that is, the right-hand side) where polymersomes have a higher concentration of PEO-PDPA, we observed a similar arrangement of 2D micelles, only this time the micelles seemed to be smaller (about 5.5 nm) and their cores were composed of PMPC chains instead of PEO. Also, this phase shows some sort of ordered arrangement of the micelles onto the surface. In the central part of the diagram, polymersomes have a surface topology, with the black and white domains well connected between each other, forming a bicontinuous pattern. Using the geometrical parameters calculated in Fig. 1, we can define the three phases. For micellar 1, the average size of 7 nm corresponds to about eight times the PDPA radius, suggesting a regular hexagonal packing of 6 PEO-PDPA diblock surrounded by 12 PEO-PDPA-PMPC triblocks. For micellar 2, the domain size of 5.5 nm corresponds to about two times the PMPC radius, suggesting a hexagonal array comprising one PMPC-PDPA copolymer surrounded by six PEO-PDPA-PMPC triblocks. We observe that the bicontinuous phase is the arrangement showing more efficient packing in 2D, and this explains why their corresponding topologies are indeed the most symmetrical ones. However, in all three phases, the presence of the PEO-PDPA copolymers clearly aids the self-assembly of the triblock facilitating the formation of quite controlled patterns.

## CONCLUSIONS

We demonstrated here that polymersomes can be constructed with complex surface topologies creating the necessary conditions for a membrane-confined self-assembly. This is achieved by introducing an interfacial energy in the system, created by the interaction of two hydrophilic blocks forced to share the same structure, the polymersome, and a triblock copolymer bearing the same two polymers that act as stabilizers. We show that this judicious copolymer mixture can self-assemble into domains with geometries and patterns that recall those formed by micelles in three dimensions. Finally, we propose more complex designs of supramolecular structures as a new approach in mimicking biological units, such as viruses and vesicles.

## MATERIALS AND METHODS

### Materials

2-(Methacryloyloxy)ethyl phosphorylcholine monomer (MPC; 99.9% purity) was donated by Biocompatibles UK Ltd. Anhydrous ethanol (99%), anhydrous methanol (≥99.8%), 2-(diisopropylamino)ethyl methacrylate (DPA), copper(I) bromide [Cu(I)Br; 99.999%], 2,2′-bipyridine (bpy; 99%), tris(2-carboxyethyl)phosphine hydrochloride (TCEP; ≥98.0%), dry triethylamine, and PTA were purchased from Sigma Aldrich UK. The silica gel 60 (0.063 to 0.200 μm) used to remove the spent atom transfer radical polymerization (ATRP) catalyst CuBr was purchased from E. Merck. High-performance liquid chromatography–grade dichloromethane and methanol were purchased from Fisher Scientific. All of the above were used as received. Phosphate-buffered saline (PBS) was prepared from tablets obtained from Oxoid. Semipermeable cellulose dialysis tubing (Spectra/Por 6 MWCO 1000) was purchased from Fisher Scientific.

### Synthesis of PMPC-PDPA

We slightly altered a previously published procedure to synthesize linear PMPC_25_-PDPA_70_ diblock (*18*). A solution of morpholinoethyl-bromoisobutyric acid ester (described previously) (*18*) (0.190 g, 0.00068 mol, 1 eq) was placed in a round-bottomed flask before the addition of MPC (5.000 g, 0.017 mol, 25 eq). The mixture was dissolved in 5 ml of ethanol, and further purged with nitrogen for 30 min and heated to 30°C. Then, a mixture of bpy (0.223 g, 0.00142 mol, 2 eq) and Cu(I)Br (0.097 g, 0.00068 mol, 1 eq) was added under a constant nitrogen flow. The mixture was stirred for 60 min to yield a highly viscous brown substance and sampled for nuclear magnetic resonance (NMR) to estimate conversion (gave full conversion). A solution of DPA (12.27 g, 0.0576 mol, 85 eq) in 13 ml of ethanol was prepared and purged with nitrogen for 60 min in a separate flask. Then, the DPA solution was added to the polymerization mixture, and the reaction mixture was purged for another 10 min and then left overnight at 30°C. After 18 hours, ^1^H NMR analysis confirmed that the conversion was >99%, and the reaction was opened to the atmosphere and diluted with ethanol. The solution gradually turned green, indicating oxidation of the copper-based catalyst system. The green solution was passed through silica using ethanol and partially evaporated to give an opaque solution. The solution was then dialyzed [molecular weight cutoff (MWCO), 1000 daltons] against dichloromethane (two times), methanol (1:1) (two times), and water (two times) for 8 to 14 hours for each dialysis cycle. The polymer was first freeze-dried and then dried at 120°C for 2 hours under vacuum. Then, the polymer was again dried for 24 hours at 90°C under vacuum (13.3 g, 77% yield). ^1^H NMR (CDCl_3_/MeOD, 3:1) composition was PMPC_25_-PDPA_72_.

### Synthesis of PEO macroinitiator

We adopted a previously published procedure by Voit *et al.* (*28*). Here, 10 g (0.002 mol) of PEO_45_-OH was dried in a flask under vacuum at 70°C for 30 min. The flask was flushed with nitrogen before 20 ml of dry tetrahydrofuran (THF) was added (0.92 g, 0.004 mmol). 2-Bromoisobutyric acid bromide was dissolved in 3 ml of dry THF before adding to the solution. The flask was cooled with ice, and 0.303 g (0.003 mol) of dry triethylamine was added to the existing solution. The turbid mixture was stirred for 40 hours at room temperature. The final macroinitiator was then dialyzed (MWCO, 1 kD) against methanol (two times) and deionized water (two times) before being freeze-dried (74% yield). ^1^H NMR (CDCl_3_) was performed as described and according to previously published ratios to PEO_45_-Br (*28*). A similar procedure was used for the synthesis of PEO_23_-Br macroinitiator.

### Synthesis of PEO-PDPA

We adopted a previously published procedure (*29*). A solution of PEO_23_-Br (0.500 g, 0.00010 mol, 1 eq) was put in a round-bottomed flask and dissolved in 3 ml of ethanol, and DPA (2.29 g, 0.0108 mol, 20 eq) was added. The mixture was purged with nitrogen for 30 min and heated at 30°C. Then, a mixture of bpy (0.032 g, 0.00022 mol, 2 eq) and Cu(I)Br (0.014 g, 0.00011 mol, 1 eq) was added under a constant nitrogen flow. The mixture was stirred overnight at 30°C to yield a highly viscous brown substance, which was sampled for NMR to estimate conversion (gave full conversion). The reaction was opened to the atmosphere and diluted with ethanol. The solution gradually turned green, indicating oxidation of the copper-based catalyst system. The green solution was passed through silica using ethanol and partially evaporated to give an opaque solution. The solution was dialyzed (MWCO, 1000 daltons) against dichloromethane (two times), methanol (1:1) (two times), and water (two times) for 8 to 14 hours for each dialysis cycle. The polymer was freeze-dried under vacuum and dried at 120°C for 2 hours under vacuum before drying for another 24 hours at 90°C, also under vacuum. (2.83 g, 98% yield). ^1^H NMR (CDCl_3_) was performed as described and according to previously published ratios to PEO_23_-PDPA_17_.

### Synthesis of PEO-PDPA-PMPC

Linear triblock PEO_45_-PDPA_60_-PMPC_12_ was synthesized by ATRP, following an adapted procedure of Blanazs and coworkers (*26*). Briefly, the PEO-Br macroinitiator was first synthesized according to the procedure described above. The purified compound (135 mg, 0.063 mmol, 1 eq) was subjected under vacuum for 30 min. In another round-bottomed flask, DPA monomer (808 mg, 3.89 mmol, 60 eq) was diluted in ethanol to slightly decrease its viscosity, and purged with nitrogen for 30 min. The DPA solution was subsequently transferred to the PEO-Br solution, and the mixture was further purged with nitrogen at 30°C. Then, CuBr (9.06 mg, 0.063 mmol, 1 eq) and bpy (19.75 mg, 0.12 mmol, 2 eq) were weighed off and added as solids in this mixture, and the polymerization was carried out for at least 3 hours, until any DPA monomer could be detected by NMR. The MPC monomer (224 mg, 0.76 mmol, 12 eq) was then solubilized in ethanol and purged for 30 min with nitrogen. This solution was added to the reaction mixture and left to polymerize overnight. The highly viscous brown raw product was checked by ^1^H NMR (CDCl_3_/MeOD, 3:1) for 90% DPA and 99% MPC conversion and then opened to the atmosphere to dilute in ethanol. The solution gradually turned green, indicating oxidation of the copper-based catalyst system. The green solution was passed through silica using ethanol and evaporated partially to give an opaque solution. The solution was dialyzed (MWCO, 1000 daltons) against dichloromethane (two times), methanol (1:1) (two times), and water (two times) for 8 to 14 hours for each dialysis cycle. The polymer was freeze-dried under vacuum and dried at 120°C for 2 hours under vacuum before drying for another 24 hours at 90°C, also under vacuum (894 mg, 87% yield). The purified triblock was analyzed by ^1^H NMR (fig. S1) and gel permeation chromatography (GPC) (fig. S2); ^1^H NMR (600 MHz; CDCl_3_/MeOD, 3:1; ppm) δ: 0.66 [broad peak **l**, 3H, −(CH_3_)], 0.79 (doublet **a**, 12H, C**H_3_**−CH−C**H_3_**), 1.0 (doublet **a**, 12H, C**H_3_**–CH–C**H_3_**), 1.50 to 1.90 (broad peaks **g**, backbone), 2.42 (broad peak **b**, 2H, CH3−C**H**−CH3), 2.78 (broad peak **c**, H, −O−CH_2_−C**H_2_**−N−), 3.09 (singlet **f**, 9H, C**H_3_**−N−), 3.40 (broad peak **e**, 4H, −O−C**H_2_**−C**H_2_**−O−), 3.48 (broad peak **h**, 2H, −P−O−CH_2_−C**H_2_**−N−), 3.62 (broad peak **d**, 2H, −O−CH_2_−C**H_2_**−N−), 3.73 (broad peak **j**, 2H, −O−CH_2_−C**H_2_**−O−P−), 3.79, 3.94 (broad peak **i**, 2H, −P−O−C**H_2_**−CH_2_−N−), 4.03 (broad peak **k**, 2H, −O−C**H_2_**−CH_2_−O−P−), and composition PEO_45_-PDPA_60_-PMPC_12_. The GPC trace in 0.25% trifluoroacetic acid (TFA) aqueous solution gave a retention volume of 8.04 ml and a polydispersity index (PDI) of 1.13.

### Gel permeation chromatography

GPC was carried out using a Malvern Viscotek GPC system (Malvern Instruments) and a Novema Max 100Å Column with a Novema Max Guard Column (both from PSS Polymer) with 0.25 (v/v) TFA in water as an eluent or a ResiPore 100Å Column with a ResiPore Guard Column (Agilent Technologies) with a chloroform/methanol (3:1) eluent.

### NMR spectroscopy

NMR spectroscopy was carried out on a Bruker AV 600 spectrometer (14.1-T magnetic field strength, operating at 600 MHz for ^1^H NMR and at 125 MHz for ^13^C NMR). Water was used from a TKA water purification system (Thermo Scientific).

### Patchy polymersome formation

Binary and ternary copolymer mixtures were prepared by mixing PMPC_25_-PDPA_70_ with PEO_45_-PDPA_60_-PMPC_12_ and PMPC_25_-PDPA_70_ with PEO_45_-PDPA_60_ and PMPC_12_-PEO_23_-PDPA_15_ at various molar ratios. Nanometer-sized polymersomes were formed by the film rehydration method. The polymer mixtures were dissolved in 2:1 (v/v) chloroform/methanol at a total copolymer concentration of 10 mg/ml in organic solvent. The solution was placed in a vacuum oven at 40°C and left overnight to evaporate the organic solvent. A copolymer dried thin film was formed on the sample vial surface. Rehydration of the PMPC_25_-PDPA_70_ /PEO_45_-PDPA_60_-PMPC_12_ and PMPC_25_-PDPA_70_ / PEO_45_-PDPA_60_-PMPC_12_ /PEO_23_-PDPA_15_ film was performed using 0.1 M PBS (pH 7.4) at a copolymer concentration of 5 mg/ml. The aqueous dispersions were stirred with a magnetic stirrer at 2000 rpm for 2 weeks at room temperature. The polymersome solution was then centrifuged for 15 min at 500 relative centrifugal force (rcf), and then for 5 min at 2000 rcf using an Eppendorf microcentrifuge. Centrifugation was performed to purify the solution and narrow down polymersome sizes, as large and small particles remained in the pellet and supernatant, respectively.

### TEM imaging

Conventional TEM imaging was performed using an FEI Tecnai G2 Spirit TEM microscope at 80 kV equipped with an Orius SC1000 camera. The polymersomes were stained using a PTA solution at 0.75% (w/v). PTA at 10% (w/v), supplied by Sigma-Aldrich, was used. The solution was prepared by dissolving 37.5 mg of PTA in boiling distilled water (5 ml). The pH was adjusted to 7.0 by adding a few drops of 5 M NaOH under continuous stirring. The PTA solution was then filtered through a 0.2-μm filter.

Copper grids were glow-discharged for 40 s to render them hydrophilic. Then, 5 μl of polymersome/PBS dispersion (diluted 10-fold; concentration, 0.5 mg/ml) was deposited onto the grids for 1 min. Then, the grids were blotted with filter paper and immersed in the PTA staining solution for 5 s for negative staining. The grids were blotted again and dried under vacuum for 1 min.

### Image analysis

The average spacing of the domains formed on the PMPC-PDPA/PEO-PDPA-PMPC polymersome surface was calculated as a function of triblock concentration, and is shown in fig. S1. To perform such calculations, we used Matlab because it allowed for a good statistical analysis. Four different polymersomes were analyzed for every prepared formulation, and the number of patches used for the calculations ranged from 50 to 100 for each polymersome. The Matlab script stored the *x* and *y* coordinates of the domain center of mass. The script then calculated the distances (pixels) between all the points chosen using the Pythagoras’ theorem. That is, if three points were chosen, namely, A, B, and C, Matlab would calculate the distance of A from B and C, the distance of B from A and C, and the distance of C from A and B. For every single set of distances, Matlab would save only the shortest distance between two points. For example, if A is closer to B than it is to C, only the distance of A from B would be saved for later calculations. Final distances were converted to nanometers using the pixel to nanometer ratio previously defined. Once all the distances were calculated, Matlab averaged the distances and calculated the SD. This method yielded results that were in very good agreement with the distances manually measured by ImageJ software. The resulting calculations are shown in fig. S1. As shown in Fig. 2, at 10% triblock concentration, the domains were formed by the PEO chains immersed on a PMPC matrix (white unstained PEO versus black stained PMPC). These domains had sizes ranging from 6 to 10 nm, with most of them having a circular shape. This trend was observed for low triblock concentrations ranging from 1 to 10%. Accordingly, the domain spacing was measured for the white PEO domains. At higher concentrations, the elongated conformation became dominant, and at triblock concentrations between 40 and 80%, the domains merged, forming a bicontinuous pattern. In these concentrations, the spacing was measured for both PEO and PMPC domains. Unsurprisingly, the resulting spacing was symmetrical for both polymers. Finally, at 90% concentration, the black PMPC domains seemed to assume a discrete shape in a PEO white matrix, suggesting some sort of symmetrical arrangement. For 90 and 100% triblock concentrations, the spacings were measured for the PMPC patches. The average domain spacing, as a function of triblock concentration, is shown in Fig. 2.

### Patchy polymersome simulation

A coarse-grained model of the copolymers diffusing on a spherical surface was devised. Each PMPC-PDPA copolymer was represented as a single bead of mass *m*_1_ representing the PMPC solvent-exposed chain. Assuming that the PEO-PDPA-PMPC copolymer adopts a conformation where both the PEO and PMPC chains are solvent-exposed (Fig. 1D), we can represent the copolymer using two connected beads of masses *m*_2_ and *m*_3_. To emulate the interactions of the PMPC and PEO beads, we assumed a 12-6 Lennard-Jones potential for beads of the same kind (that is, PMPC/PMPC and PEO/PEO), with parameters $E_{1}$,*r*_1_ and $E_{2}$,*r*_2_, respectively. Beads of different kinds (PMPC/PEO) repel each other with a repulsive potential

$$U(r)={\left(\frac{r_{1}}{r}\right)}^{12}$$

where *r* is the distance between beads. The maximum distance between the two PEO and PMPC beads, representing a single PEGO-PDPA-PMPC copolymer, is fixed at *d*_max_, and it is enforced with a potential *U*_*R*_(*r*) = 0 for *r* < *d*_max_ and *U*_*R*_(*r*) = *k*(*r* − *d*_max_)^2^ for *r* > *d*_max_. The diffusion of the beads was constrained on a polymersome surface of radius *R*. Assuming values loosely connected to the corresponding physical system, due to the level of coarse-graining of the parameters, leads to *m*_1_ = 2313 uma, *m*_2_ = 774 uma, *m*_3_ = 993 uma, $E_{1}$ = 1 kJ mol^−1^, *r*_1_ = 0.3 nm, *r*_2_ = 0.2 nm, *k* = 10^4^ kJ mol^−1^ nm^−2^, *d*_max_ = 1.5 nm, and *R* = 19.2 nm. The distances were chosen to represent approximately 1/10 of the observed separation distance between blocks, and the beads masses to represent 1/10 of the corresponding copolymer mass

$$\begin{array}{c} m_{1}=\frac{1}{10}(25m_{\text{PMPC}}+74m_{\text{PDPA}})\\ m_{2}=\frac{1}{10}(45m_{\text{PEO}}+30m_{\text{PDPA}})\\ m_{3}=\frac{1}{10}(12m_{\text{PMPC}}+30m_{\text{PDPA}}) \end{array}$$

## Supplementary Material

http://advances.sciencemag.org/cgi/content/full/2/4/e1500948/DC1

## SUPPLEMENTARY MATERIALS

Supplementary material for this article is available at http://advances.sciencemag.org/cgi/content/full/2/4/e1500948/DC1 fig. S1. ^1^H NMR spectrum of PEO-PDPA-PMPC triblock copolymer in CDCl_3_/MeOH (3:1); composition: PEO_45_-PDPA_60_-PMPC_12_ (relative integration of protons e versus b/c and h). fig. S2. GPC trace of PEO-PDPA-PMPC triblock copolymer in 0.25% TFA aqueous solution, PDI = 1.13, superimposed to PEO_45_-PDPA_60_. fig. S3. Representative size distribution of PMPC-PDPA polymersomes containing different amounts of PMPC-PDPA-PEO triblock copolymers measured by dynamic light scattering. fig. S4. Low-magnification image of PMPC-PDPA/PMPC-PDPA-PEO binary mixture (90:10). fig. S5. Low-magnification image of PMPC-PDPA/PMPC-PDPA-PEO binary mixture (80:20). fig. S6. Low-magnification image of PMPC-PDPA/PMPC-PDPA-PEO binary mixture (60:40). fig. S7. Low-magnification image of PMPC-PDPA/PMPC-PDPA-PEO binary mixture (40:60). fig. S8. Low-magnification image of PMPC-PDPA/PMPC-PDPA-PEO binary mixture (10:90). fig. S9. Low-magnification image of PMPC-PDPA/PMPC-PDPA-PEO/PEO-PDPA ternary mixture (10:80:10). fig. S10 (A) Low-magnification image of PMPC-PDPA/PMPC-PDPA-PEO/PEO-PDPA ternary mixture (10:60:30). (B) Low-magnification image of PMPC-PDPA/PMPC-PDPA-PEO/PEO-PDPA ternary mixture (10:30:60). fig. S11. Low-magnification image of PMPC-PDPA/PMPC-PDPA-PEO/PEO-PDPA ternary mixture (60:30:10).
