# Supplementary material for: Molecular engineering of polymersome surface topology
Source: Sci Adv. 2016 Apr 15;2(4):e1500948. doi: 10.1126/sciadv.1500948 (PMC4846435; doi:10.1126/sciadv.1500948)
Supplement: http://advances.sciencemag.org/cgi/content/full/2/4/e1500948/DC1 [file supp_2_4_e1500948__index.html]

Science Advances | Science Advances

## Supplementary Materials

**This PDF file includes:**

- fig. S1. 1H NMR spectrum of PEO-PDPA-PMPC triblock copolymer in CDCl3/MeOH (3:1); composition: PEO45-PDPA60-PMPC12 (relative integration of protons e versus b/c and h).
- fig. S2. GPC trace of PEO-PDPA-PMPC triblock copolymer in 0.25% TFA aqueous solution, PDI = 1.13, superimposed to PEO45-PDPA60.
- fig. S3. Representative size distribution of PMPC-PDPA polymersomes containing different amounts of PMPC-PDPA-PEO triblock copolymers measured by dynamic light scattering.
- fig. S4. Low-magnification image of PMPC-PDPA/PMPC-PDPA-PEO binary mixture (90:10).
- fig. S5. Low-magnification image of PMPC-PDPA/PMPC-PDPA-PEO binary mixture (80:20).
- fig. S6. Low-magnification image of PMPC-PDPA/PMPC-PDPA-PEO binary mixture (60:40).
- fig. S7. Low-magnification image of PMPC-PDPA/PMPC-PDPA-PEO binary mixture (40:60).
- fig. S8. Low-magnification image of PMPC-PDPA/PMPC-PDPA-PEO binary mixture (10:90).
- fig. S9. Low-magnification image of PMPC-PDPA/PMPC-PDPA-PEO/PEO-PDPA ternary mixture (10:80:10).
- fig. S10. (A) Low-magnification image of PMPC-PDPA/PMPC-PDPA-PEO/PEO-PDPA ternary mixture (10:60:30). (B) Low-magnification image of PMPC-PDPA/PMPC-PDPA-PEO/PEO-PDPA ternary mixture (10:30:60).
- fig. S11. Low-magnification image of PMPC-PDPA/PMPC-PDPA-PEO/PEO-PDPA ternary mixture (60:30:10).

Download PDF

**Files in this Data Supplement:**

- Adobe PDF - 1500948\_SM.pdf
