# Supplementary material for: Molecular engineering of polymersome surface topology
Source: Sci Adv. 2016 Apr 15;2(4):e1500948. doi: 10.1126/sciadv.1500948 (PMC4846435; doi:10.1126/sciadv.1500948)
Supplement: http://advances.sciencemag.org/cgi/content/full/2/4/e1500948/DC1 [file 1500948_SM.pdf]

Science Advances

AAAS

advances.sciencemag.org/cgi/content/full/2/4/e1500948/DC1

Supplementary Materials for

# Molecular engineering of polymersome surface topology

Lorena Ruiz-Perez, Lea Messager, Jens Gaitzsch, Adrian Joseph, Ludovico Sutto, Francesco Luigi Gervasio, Giuseppe Battaglia

Published 15 April 2016, *Sci. Adv.* **2**, e1500948 (2016)
DOI: 10.1126/sciadv.1500948

**This PDF file includes:**

- fig. S1. $^1$H NMR spectrum of PEO-PDPA-PMPC triblock copolymer in $CDCl_3$/MeOH (3:1); composition: $PEO_{45}$-$PDPA_{60}$-$PMPC_{12}$ (relative integration of protons e versus b/c and h).
- fig. S2. GPC trace of PEO-PDPA-PMPC triblock copolymer in 0.25% TFA aqueous solution, PDI = 1.13, superimposed to $PEO_{45}$-$PDPA_{60}$.
- fig. S3. Representative size distribution of PMPC-PDPA polymersomes containing different amounts of PMPC-PDPA-PEO triblock copolymers measured by dynamic light scattering.
- fig. S4. Low-magnification image of PMPC-PDPA/PMPC-PDPA-PEO binary mixture (90:10).
- fig. S5. Low-magnification image of PMPC-PDPA/PMPC-PDPA-PEO binary mixture (80:20).
- fig. S6. Low-magnification image of PMPC-PDPA/PMPC-PDPA-PEO binary mixture (60:40).
- fig. S7. Low-magnification image of PMPC-PDPA/PMPC-PDPA-PEO binary mixture (40:60).
- fig. S8. Low-magnification image of PMPC-PDPA/PMPC-PDPA-PEO binary mixture (10:90).
- fig. S9. Low-magnification image of PMPC-PDPA/PMPC-PDPA-PEO/PEO-PDPA ternary mixture (10:80:10).
- fig. S10. (A) Low-magnification image of PMPC-PDPA/PMPC-PDPA-PEO/PEO-PDPA ternary mixture (10:60:30). (B) Low-magnification image of PMPC-PDPA/PMPC-PDPA-PEO/PEO-PDPA ternary mixture (10:30:60).
- fig. S11. Low-magnification image of PMPC-PDPA/PMPC-PDPA-PEO/PEO-PDPA ternary mixture (60:30:10).

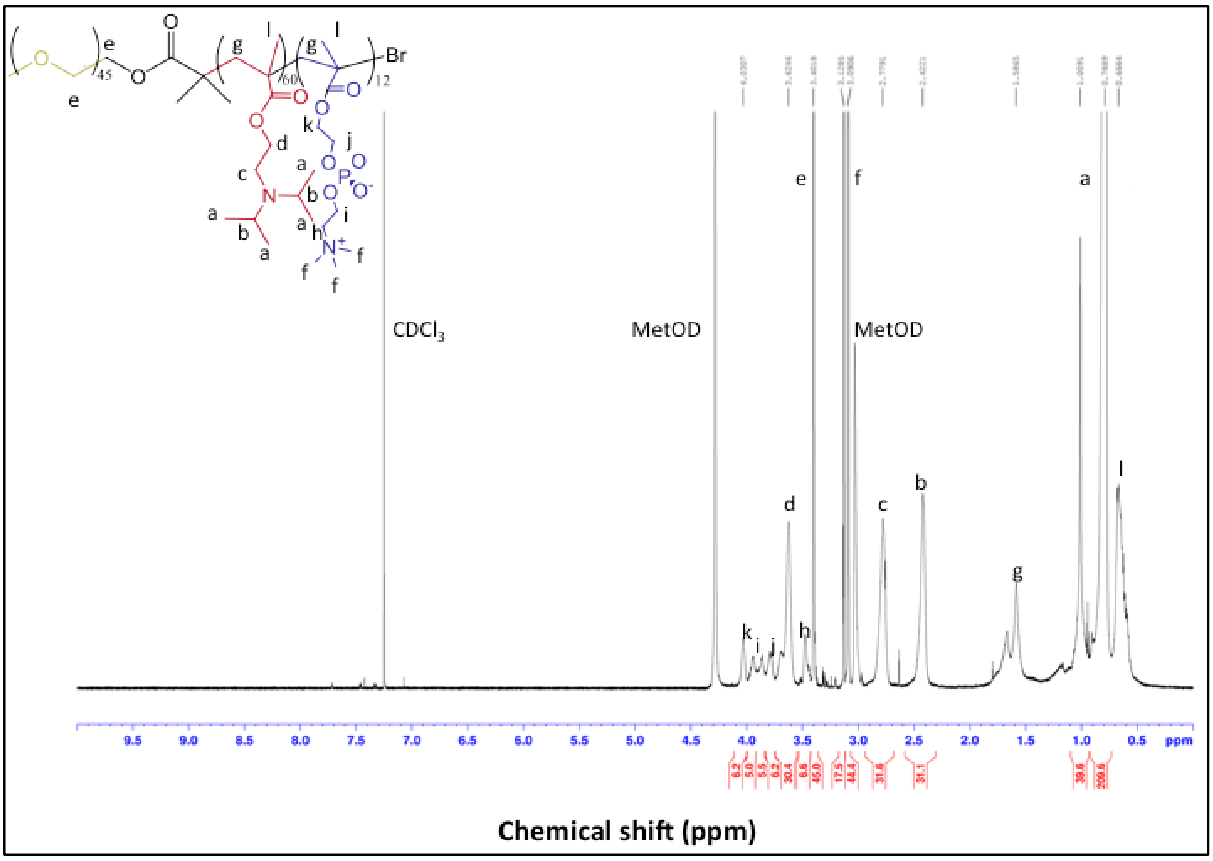


fig. S1. $^{1}$H NMR spectrum of PEO-PDPA-PMPC triblock copolymer in $CDCl_3$/MetOH (3:1); composition: $PEO_{45}$-$PDPA_{60}$-$PMPC_{12}$ (relative integration of protons e versus b/c and h).

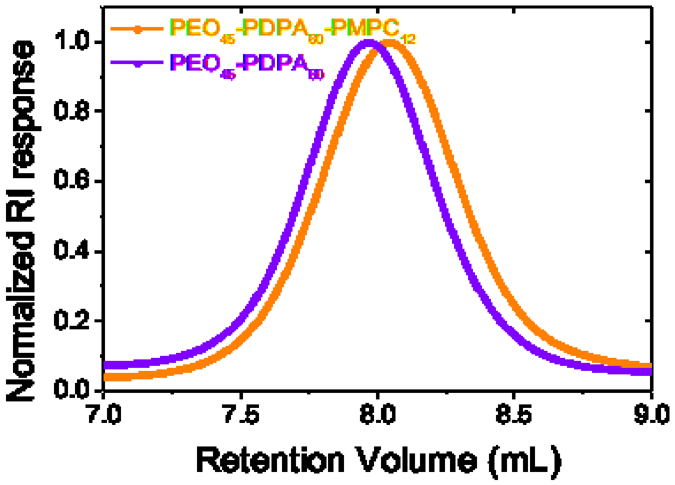


fig. S2. GPC trace of PEO-PDPA-PMPC triblock copolymer in 0.25% TFA aqueous solution, PDI = 1.13, superimposed to $PEO_{45}$-$PDPA_{60}$.

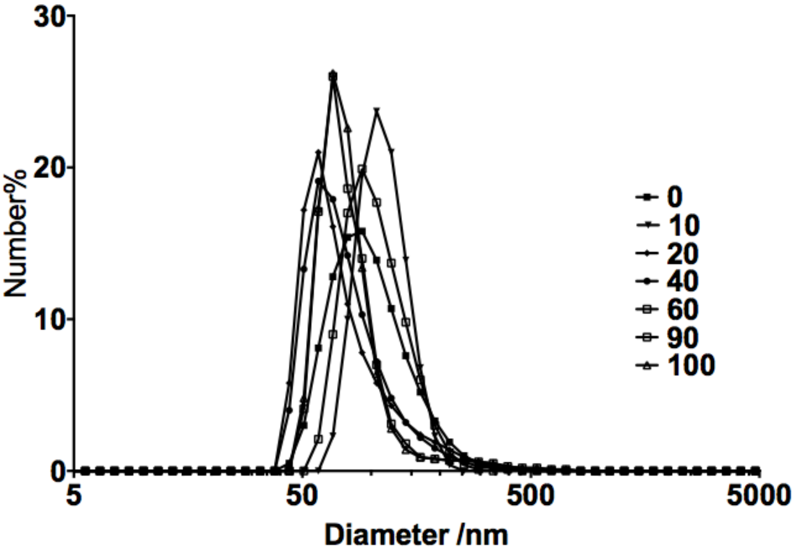


fig. S3. Representative size distribution of PMPC-PDPA polymersomes containing different amounts of PMPC-PDPA-PEO triblock copolymers measured by dynamic light scattering (DLS).

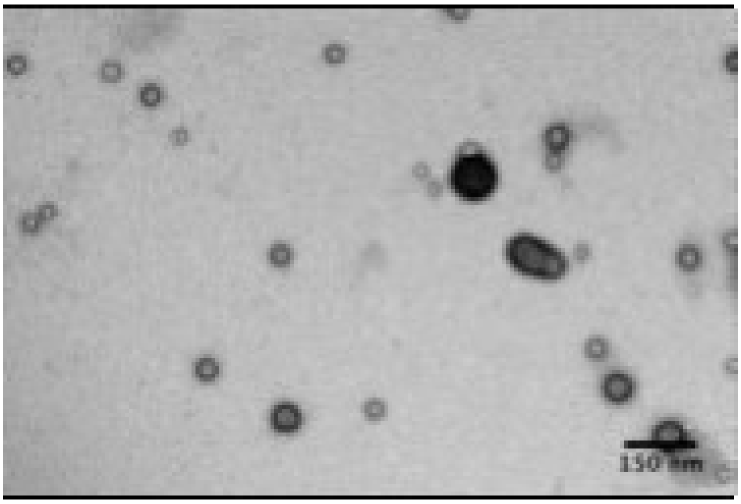


fig. S4. Low-magnification image of PMPC-PDPA/PMPC-PDPA-PEO binary mixture (90:10).

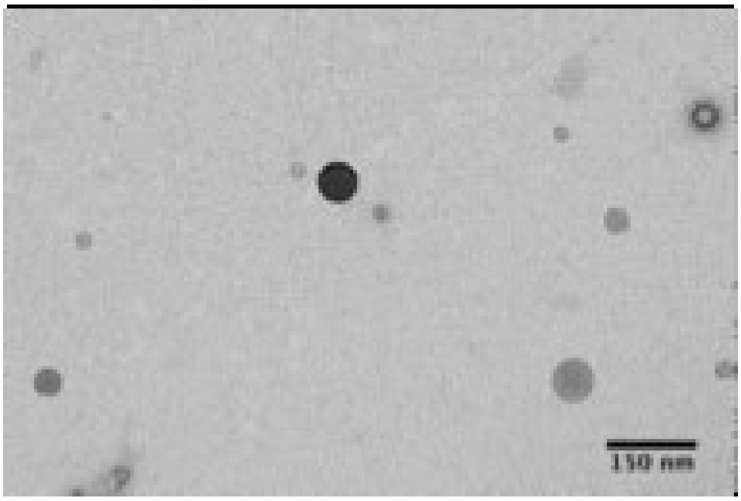


fig. S5. Low-magnification image of of PMPC-PDPA/PMPC-PDPA-PEO binary mixture (80:20).

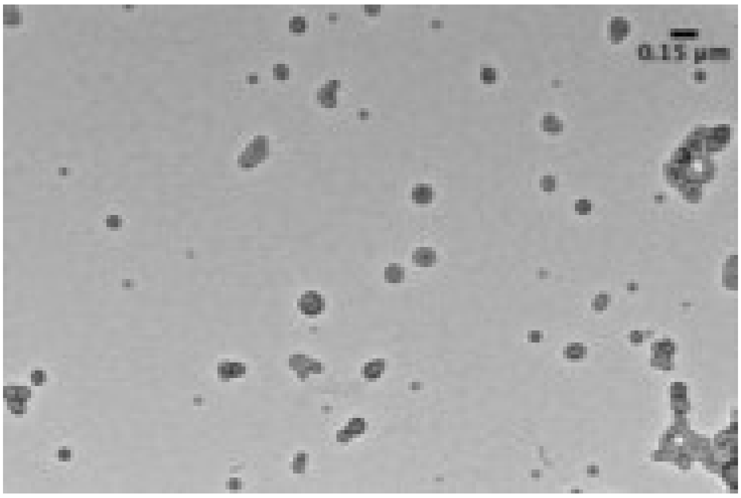


fig. S6. Low-magnification image of PMPC-PDPA/PMPC-PDPA-PEO binary mixture (60:40).

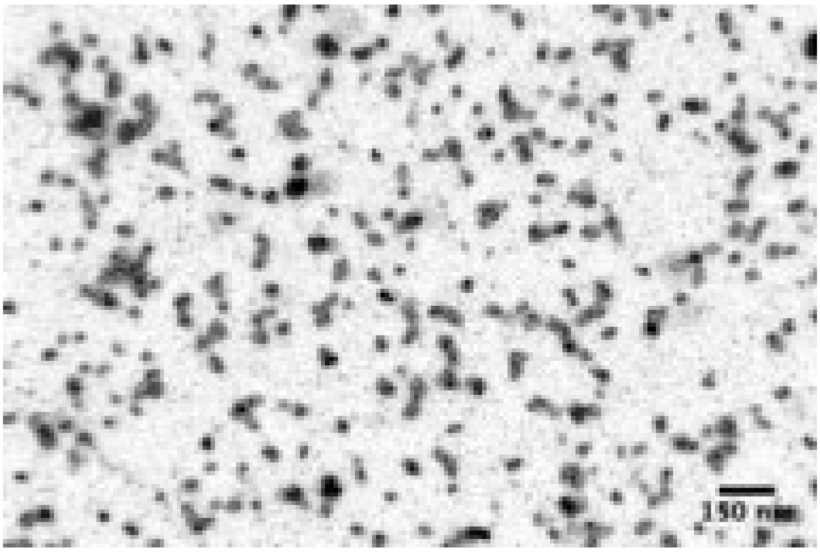


fig. S7. Low-magnification image of PMPC-PDPA/PMPC-PDPA-PEO binary mixture (40:60).

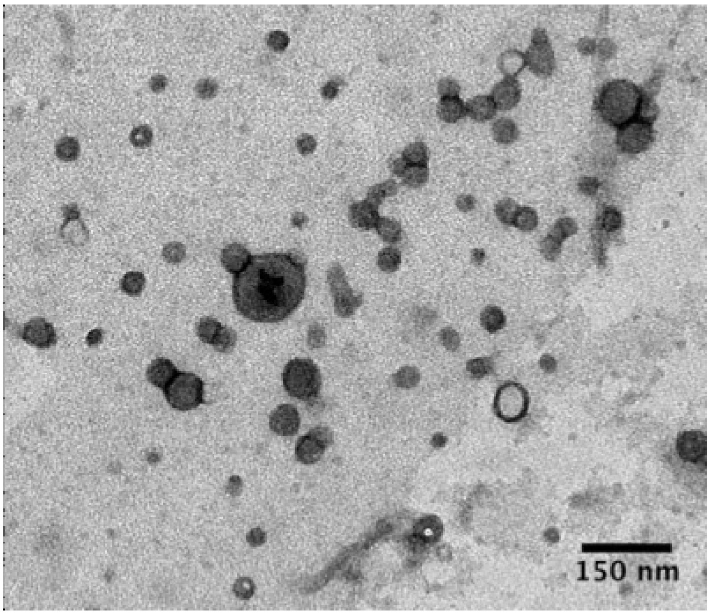


fig. S8. Low-magnification image of PMPC-PDPA/PMPC-PDPA-PEO binary mixture (10:90).

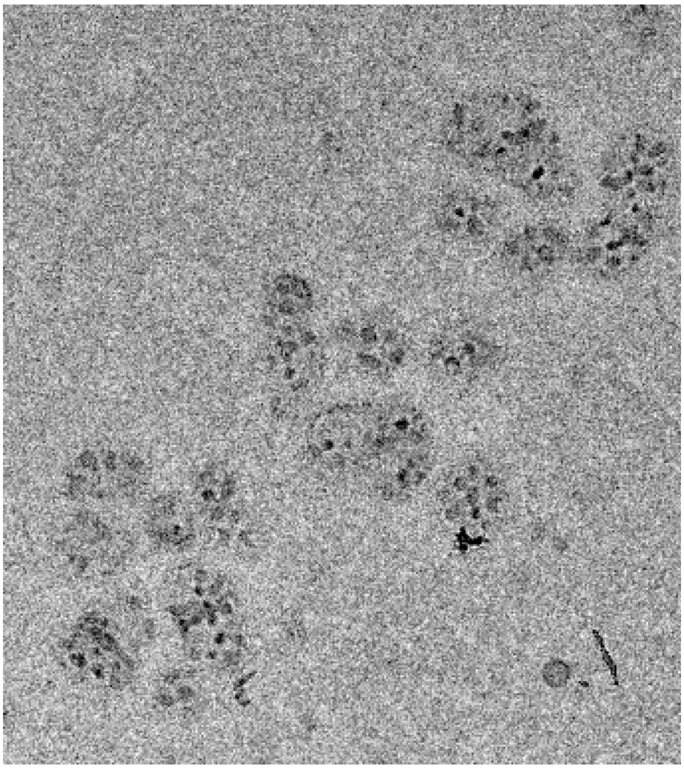

fig. S9. Low-magnification image of PMPC-PDPA/PMPC-PDPA-PEO/PEO-PDPA ternary mixture (10:80:10)

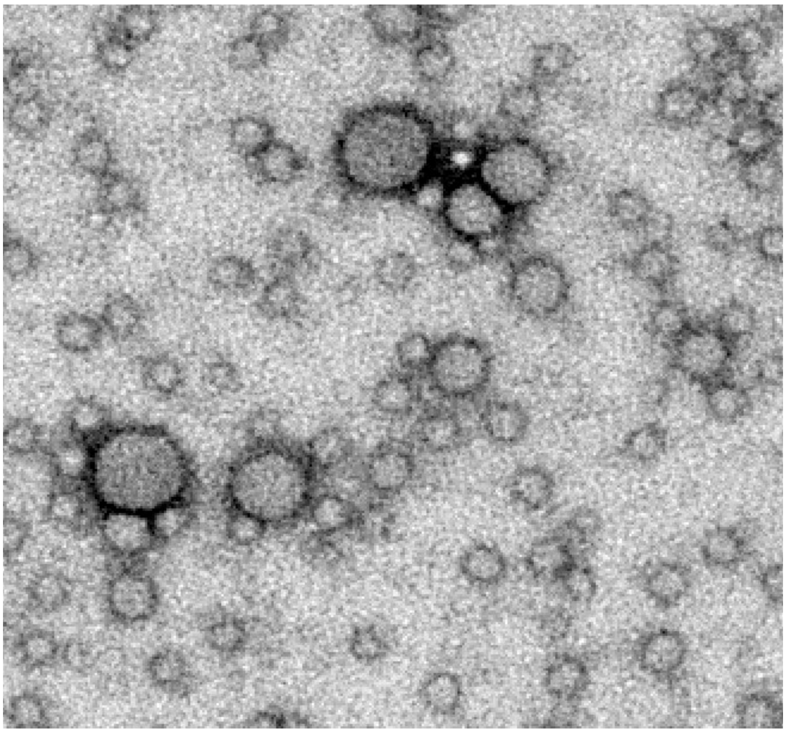

fig. S10A. Low-magnification image of PMPC-PDPA/PMPC-PDPA-PEO/PEO-PDPA ternary mixture (10:60:30).

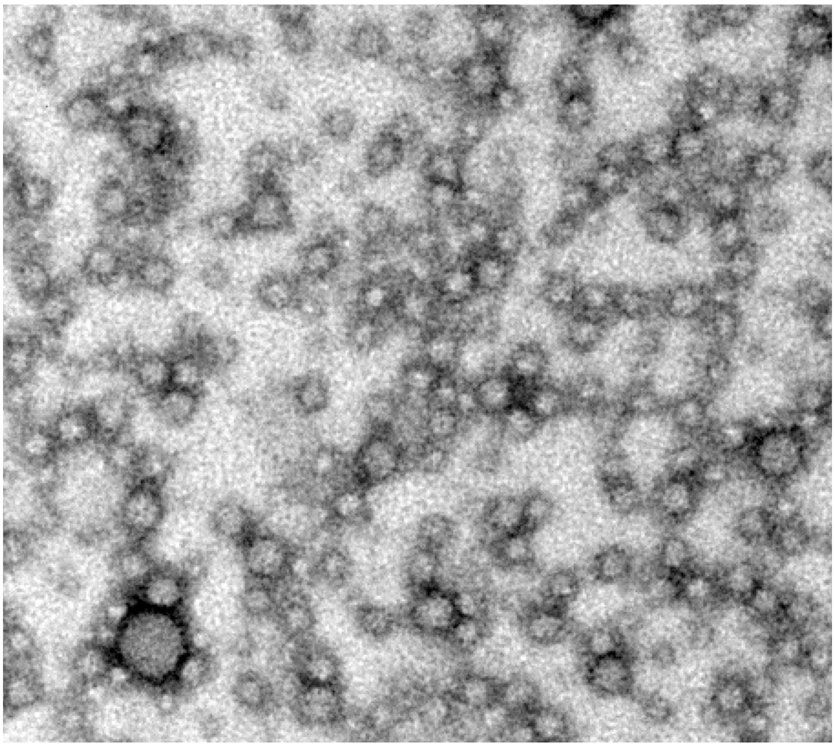

fig. S10B. Low-magnification image of PMPC-PDPA/PMPC-PDPA-PEO/PEO-PDPA ternary mixture (10:30:60).

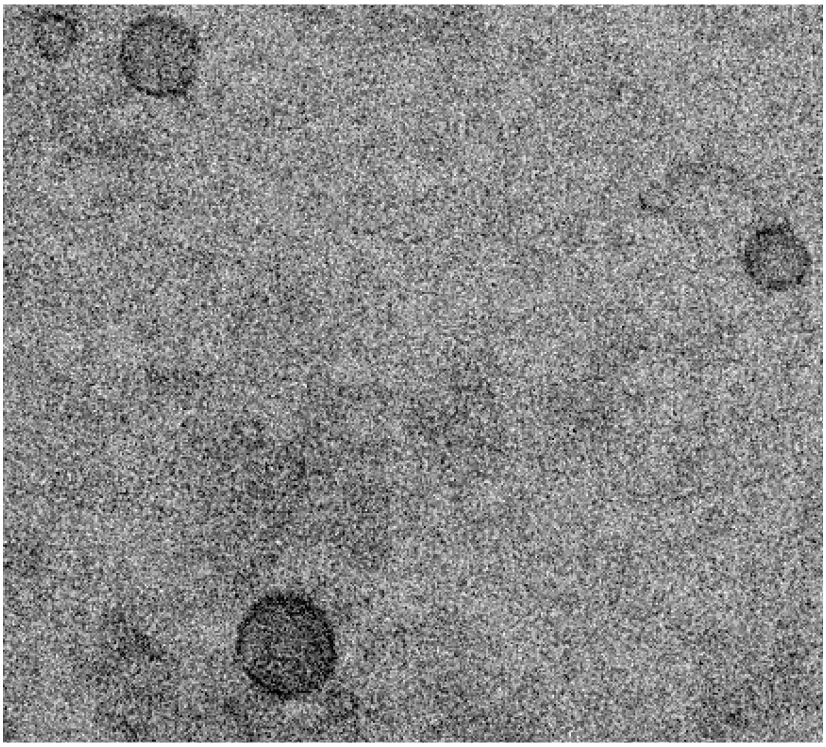

fig. S11. Low-magnification image of PMPC-PDPA/PMPC-PDPA-PEO/PEO-PDPA ternary mixture (60:30:10).
